# A Rare Case of Hemophagocytic Lymphohistiocytosis in Pregnancy With Clinical Features Resembling Acute Fatty Liver of Pregnancy

**DOI:** 10.7759/cureus.89910

**Published:** 2025-08-12

**Authors:** Atsushi Kokita, Hiromitsu Kuroda, Ryu Azumaguchi, Shintaro Suzuki, Satoshi Kazuma

**Affiliations:** 1 Department of Anesthesiology, Sapporo Medical University School of Medicine, Sapporo, JPN; 2 Department of Intensive Care Medicine, Sapporo Medical University School of Medicine, Sapporo, JPN

**Keywords:** acute liver failure (alf), aflp, hemophagocytic lymphohistiocytosis (hlh), plasma exchange therapy, coagulopathy

## Abstract

Hemophagocytic lymphohistiocytosis (HLH) during pregnancy is a rare but critical condition that is difficult to diagnose due to its complex and nonspecific presentation. We report a case of HLH in a 27-year-old woman at 32 weeks of gestation. She presented with persistent fever and liver dysfunction, leading to a suspected diagnosis of HELLP syndrome and an emergency cesarean section. However, her condition deteriorated after delivery, with the development of pancytopenia, coagulopathy, hemophagocytosis, and fatty liver. These findings raised suspicion of HLH or acute fatty liver of pregnancy (AFLP). A comprehensive diagnostic workup, including liver biopsy and genetic testing, confirmed the diagnosis of HLH. HLH during pregnancy often mimics obstetric complications such as AFLP, underscoring the importance of careful differential diagnosis.

## Introduction

Hemophagocytic lymphohistiocytosis (HLH) is a life-threatening disorder characterized by excessive immune activation and hypercytokinemia, resulting in hemophagocytosis by activated macrophages [1]. Adult-onset HLH is an extremely rare disorder, with an estimated annual incidence of approximately one in one million people [2]. Common clinical manifestations include persistent fever (100%), hepatosplenomegaly (72.9%), pancytopenia (92.9%), liver dysfunction (74.1%), and hyperferritinemia (100%) [3]. HLH is broadly classified into two categories: primary HLH, which results from genetic mutations, and secondary HLH, which is triggered by underlying conditions. In adults, secondary HLH is more prevalent and is frequently associated with infections, malignancies, or autoimmune diseases [1]. The treatment for HLH involves immunosuppressive therapy, such as corticosteroids and cyclosporine, to control hypercytokinemia. Furthermore, allogeneic hematopoietic stem cell transplantation may be performed for patients with familial, persistent, or recurrent forms of the disease [4]. HLH in critically ill patients requiring a multidisciplinary approach is associated with a poor prognosis, with mortality rates reaching 57% [5]. Early recognition and diagnosis of HLH are critical for initiating timely treatment and improving outcomes. However, diagnosis is challenging due to the condition’s nonspecific clinical features and its potential association with various underlying diseases. Lachmann et al. reported that up to 78% of HLH cases in the ICU remain undiagnosed [6].

The diagnosis of HLH in pregnancy is frequently complicated by its rarity and its clinical resemblance to common obstetric conditions like acute fatty liver of pregnancy (AFLP) and HELLP syndrome [7]. HLH must be considered an important differential diagnosis in pregnant women who present with hepatic dysfunction and thrombocytopenia, alongside AFLP and HELLP syndrome [8]. However, it is crucial to consider the possibility of HLH in patients presenting with persistent fever, cytopenia in two or more lineages, and splenomegaly. Serum ferritin is a key biomarker in the diagnosis of HLH. A ferritin level exceeding 10000 μg/L increases the diagnostic sensitivity and specificity for HLH to 90%, making it a strong indicator for the disease [9].

This report describes a case of acute liver failure in the third trimester of pregnancy, in which a comprehensive diagnostic evaluation, including liver biopsy and genetic analysis, led to the diagnosis of HLH, and the patient was successfully treated with intensive care.

## Case presentation

At 32 weeks of her second pregnancy, a 27-year-old Japanese woman presented to a local emergency facility with fever, cough, and sore throat. She was gravida 2, para 1. Her prior pregnancy was uncomplicated, and her personal and family medical histories were unremarkable. She tested negative for SARS-CoV-2 and influenza and was advised to self-monitor at home. Due to persistent high fever, she underwent further evaluation at her primary obstetric hospital one week later. Blood tests revealed markedly elevated levels of lactate dehydrogenase (LDH) at 2362 U/L and aspartate aminotransferase (AST) at 860 U/L, although neither thrombocytopenia nor hemolytic anemia was observed. HELLP syndrome was suspected, prompting her transfer to a tertiary hospital for emergency cesarean delivery. Despite the delivery, her fever persisted, and liver dysfunction progressively worsened (Table 1).

**Table 1:** Timeline of laboratory findings. This table presents serial laboratory data at critical stages of the patient’s clinical course: before delivery, upon ICU admission (ICU day 1), during the most severe phase of illness (ICU day 11), and at ICU discharge. Notably, the findings upon ICU admission, including severe hyperferritinemia, were critical markers that pointed toward a diagnosis of HLH. T-Bil: Total bilirubin, D-Bil: Direct bilirubin, AST: Aspartate aminotransferase, ALT: Alanine aminotransferase, LDH: Lactate dehydrogenase, γGT: γ-Glutamyl transpeptidase, WBC: White blood cell count, HGB: Hemoglobin, PLT: Platelet, PT-INR: Prothrombin time-international normalized ratio, APTT: Activated partial thromboplastin time, FBG: Fibrinogen.

Investigations	Normal reference range	Before delivery	ICU day 1	ICU day 11	ICU discharge
T-Bil	0.3-1.2 mg/dL	1.5	8.2	19.4	21.7
D-Bil	0-0.4 mg/dL	1.0	7	16.7	18.6
AST	13-33 U/L	860	643	255	217
ALT	6-27 U/L	105	294	148	154
LDH	119-229 U/L	2362	2507	826	701
γGT	10-47 U/L	72	204	181	171
Ferritin	10-150 ng/mL	7009	39188	24877	12725
WBC	4.0-9.0 ×10^3^ /μL	3.8	2.9	0.9	2.3
HGB	12-16.5 g/dL	9.3	9.2	8.3	8.4
PLT	150-350×10^3^ /μL	129	72	79	102
PT-INR	0.90-1.15	1.13	1.27	1.04	0.99
APTT	24-40 sec	33.2	38.3	31.2	28.8
FBG	150-340 mg/dL	230	54	35	176

**Figure 1:**
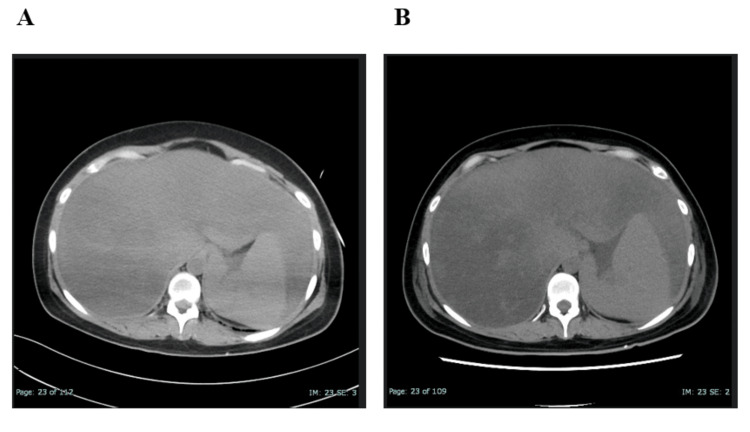
Non-contrast CT image of the upper abdomen Figure 1A was obtained at the time of cesarean section, and Figure 1B at the time of transfer to our ICU. In both images, the liver-to-spleen attenuation ratio was below 0.9, indicating fatty liver. The hepatic attenuation in Figure 1B was further reduced compared to Figure 1A. CT: Computed tomography

She subsequently developed progressive pancytopenia and coagulopathy; investigations detailed in Table 1 revealed hallmark features highly suggestive of HLH, including extreme hyperferritinemia. Bone marrow examination revealed hemophagocytosis, raising a strong suspicion of HLH. Steroid pulse therapy was initiated; however, her condition continued to deteriorate, necessitating transfer to our intensive care unit (ICU) for advanced care, including plasma exchange (PE) therapy. This procedure aims to remove pathogenic substances, such as inflammatory cytokines and bilirubin, by replacing the patient’s plasma with a replacement fluid. The decision for ICU admission was based on two primary factors. First, it was necessary to safely perform PE therapy given her high risk of bleeding due to severe coagulopathy and thrombocytopenia. Second, intensive monitoring was warranted to prepare for potential sudden deterioration, such as hypotension or respiratory failure resulting from a cytokine storm, as suggested by her rapidly worsening liver dysfunction and marked hyperferritinemia. Upon ICU admission, her vital signs were stable: clear mental status, blood pressure 106/66 mmHg, heart rate 64 bpm (beats per minute), respiratory rate 20 breaths/min, body temperature 36.5℃, and oxygen saturation 96% on room air. Computed tomography (CT) revealed fatty liver, raising concern for AFLP as a differential diagnosis (Figure 1).

**Figure 2:**
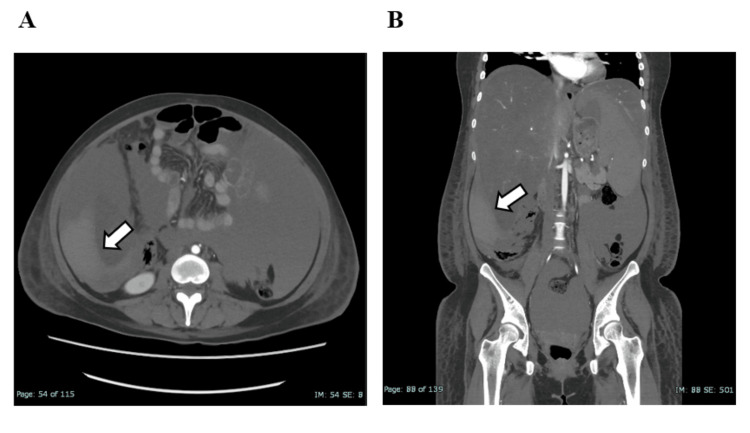
Contrast-enhanced CT after liver biopsy showing hemorrhagic ascites The images show high-density fluid consistent with hemorrhagic ascites surrounding the liver, suggestive of intra-abdominal bleeding. No obvious active extravasation was noted. CT: Computed tomography

On ICU day 2, we initiated PE therapy to manage severe liver dysfunction and coagulopathy. Due to the severity of her condition, urgent diagnostic clarification was needed to assess the indication for liver transplantation. After two sessions of PE therapy, liver function showed slight improvement, and coagulation parameters stabilized, permitting a liver biopsy on ICU day 4. On ICU day 5, she developed acute right flank pain, hypotension, and anemia. Contrast-enhanced CT revealed hemorrhagic ascites around the liver (Figure 2).

**Figure 3:**
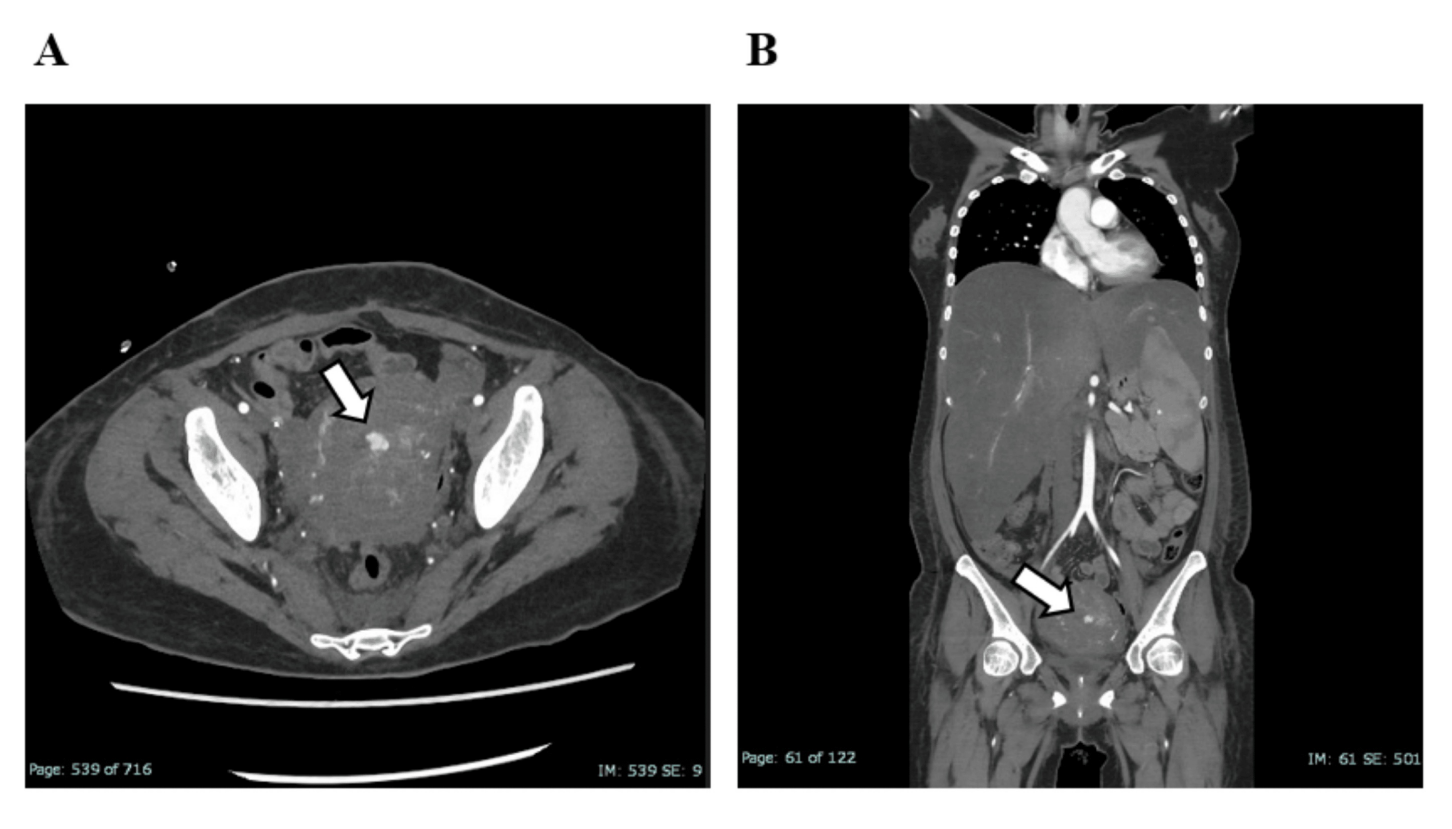
Contrast-enhanced CT showing active extravasation from the left uterine artery The images demonstrate active extravasation of contrast medium from the left uterine artery, consistent with ongoing arterial bleeding. CT: Computed tomography

Hemorrhagic shock due to intra-abdominal bleeding was diagnosed, and emergency hemostatic surgery with transfusion was performed. No active bleeding from the hepatic parenchyma was identified; however, venous bleeding was found at the hepatic flexure of the colon. Postoperatively, ischemic liver injury led to further deterioration in liver function. Liver biopsy revealed no histological features characteristic of AFLP but showed hemophagocytosis, strongly supporting a diagnosis of HLH. As her condition did not respond adequately to steroid therapy, we initiated cyclosporine on ICU day 9. On ICU day 11, worsening coagulopathy led to a decline in fibrinogen levels to 35 mg/dL, and she developed hypotension due to persistent genital bleeding. Contrast-enhanced CT revealed active extravasation from the left uterine artery (Figure 3).

**Figure 4:**
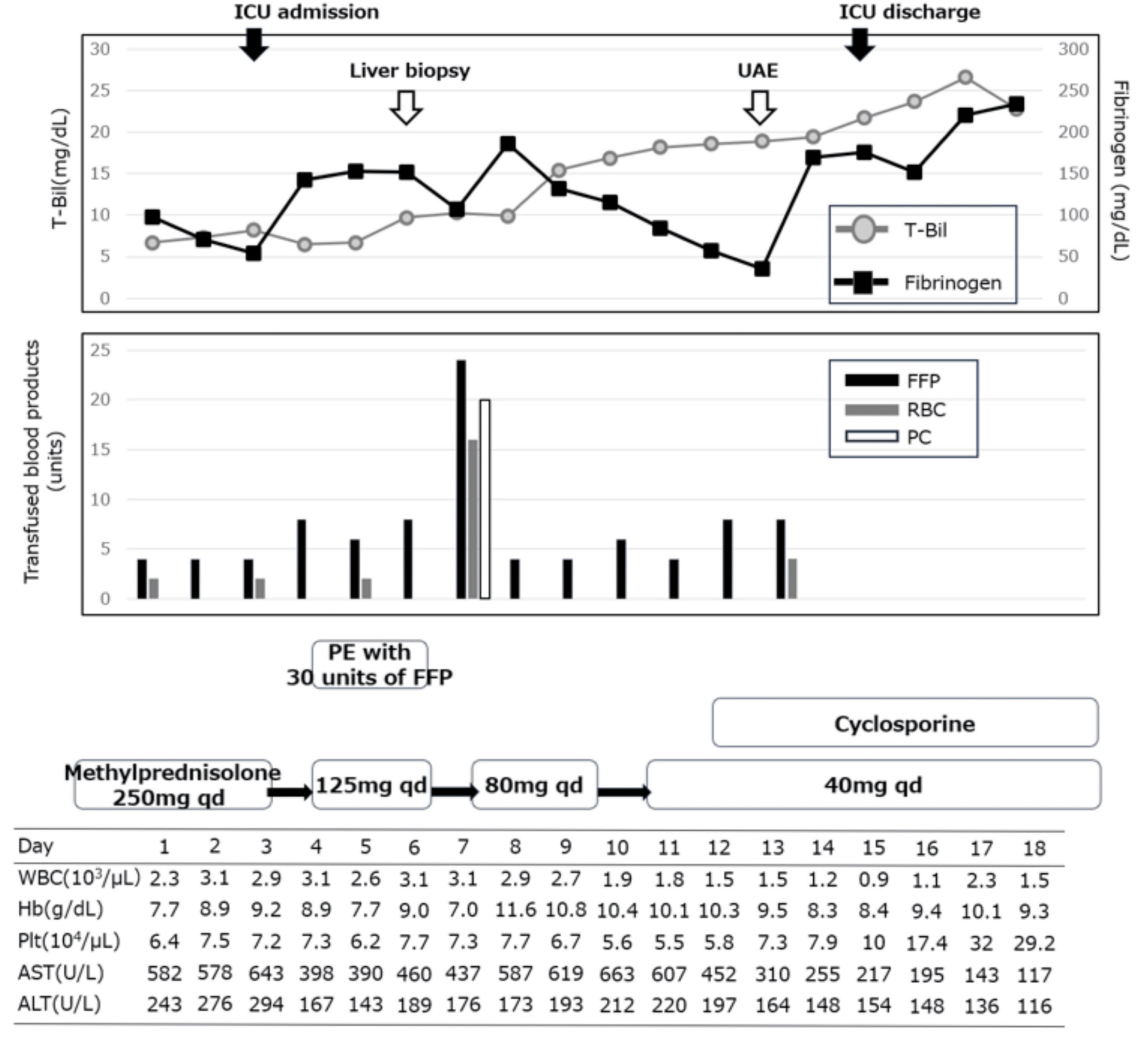
Timeline of the clinical course, laboratory results, and treatments UAE: Uterine artery embolization, T-Bil: Total bilirubin, FFP: Fresh frozen plasma, RBC: Red blood cell concentrate, PC: Platelet concentrate, PE: Plasma exchange, WBC: White blood cell, Hb: Hemoglobin, Plt: Platelet, AST: Aspartate aminotransferase, ALT: Alanine aminotransferase.

We performed uterine artery embolization (UAE) along with transfusion therapy. Although liver dysfunction persisted, her general condition stabilized. Pancytopenia and coagulopathy gradually improved, and her favorable response to cyclosporine rendered liver transplantation unnecessary. She was transferred to the general ward on ICU day 13 and to a local hospital on day 57 for continued rehabilitation (Figure 4). The newborn, followed by the pediatric department of the referring hospital, exhibited normal growth and development.

## Discussion

This report highlights the importance of differentiating between HLH and AFLP in the third trimester of pregnancy through a comprehensive approach, including histological evaluation, genetic testing, and careful assessment of the clinical course. It also underscores the necessity of advanced intensive care for managing liver failure and coagulopathy associated with HLH.

HLH during pregnancy is extremely difficult to diagnose, as its clinical presentation often mimics obstetric complications such as HELLP syndrome and AFLP [10]. The prognosis is poor, with reported maternal and fetal mortality rates reaching 22% and 40%, respectively [11]. Our case is a valuable report of a successful outcome for both mother and child despite a severe clinical course, and sharing the diagnostic and therapeutic process is of great clinical significance. In the differential diagnosis of our case, hyperferritinemia strongly supported the diagnosis of HLH. According to a review by Liu et al., only 29% of HLH cases during pregnancy had ferritin levels exceeding 10000 ng/mL [11], suggesting that our patient’s condition represented a particularly severe hyperinflammatory state. Regarding treatment, the patient was refractory to first-line steroid therapy; however, the efficacy of cyclosporine for steroid-resistant cases has been well-documented [10]. Our patient’s condition also improved following the addition of cyclosporine. While some reports suggest that termination of pregnancy can lead to remission [7], our patient’s condition deteriorated post-delivery. Therefore, the precise impact of delivery on the course of HLH during pregnancy remains unclear and warrants further investigation.

**Table 2:** Literature reports on haemophagocytic lymphohistiocytosis during pregnancy AB: Antibiotics, IVIg: Intravenous immunoglobulin, TTP: Thrombotic thrombocytopenic purpura, AFLP: Acute fatty liver of pregnancy, TMA: Thrombotic microangiopathy.

Case	Age	Period of gestation (weeks)	Clinical presentation	Ferritin (ng/mL)	Diagnostic challenge	Treatment	Maternal outcome
Cen et al. [12]	25	12	Fever, thrombocytopenia, liver dysfunction	4800	Severe pneumonia	AB, ruxolitinib, dexamethasone, etoposide	Remission
Fahad et al. [13]	30	36	Fever, thrombocytopenia, hepatosplenomegaly	2019.2	Pneumonia, viral hepatitis, HELLP syndrome	AB, acyclovir, ganciclovir, dexamethasone, IVIg	Remission
Rojas-Suarez et al. [14]	20	8	Fever, pancytopenia, liver dysfunction, coagulopathy	2985	sepsis	AB	Death
Chen et al. [9]	35	29	Fever	25766.5	Bacterial infection	AB, dexamethasone	Remission
Siddik et al. [15]	31	34	Fever, thrombocytopenia, hepatosplenomegaly	37451	Pneumonia, sepsis	AB, dexamethasone, etoposide	Death
Mostajeran et al. [16]	20	34	Fever, liver dysfunction, dyspnea,	23175	Pulmonary embolism, sepsis, postpartum endometritis	AB, methylprednisolone	Death
Potts et al. [17]	33	27	Fever, liver dysfunction, thrombocytopenia	10226	Sepsis, TTP, HELLP syndrome	AB, dexamethasone, etoposide, tacrolimus	Remission
Present case	27	32	Fever, liver dysfunction, pancytopenia, coagulopathy	39188	AFLP, HELLP syndrome, sepsis, TMA	AB, methylprednisolone, cyclosporine	Remission

We reviewed cases of HLH during pregnancy reported over the past five years and compared them with the present case (Table 2). Previous reports indicate that many cases, similar to ours, presented with non-specific symptoms such as fever, liver dysfunction, and cytopenia. The differential diagnosis commonly included sepsis and HELLP syndrome. Notably, our case clinically resembled AFLP, making the differential diagnosis particularly difficult. Furthermore, the patient’s serum ferritin level of 39188 ng/mL was the highest among the cases reviewed, likely reflecting a state of hyperinflammation. Regarding prognosis, three of the eight reviewed cases were fatal, suggesting that HLH in pregnancy is associated with a high maternal mortality rate. In many of the surviving cases, including our own, etoposide or immunosuppressive agents such as cyclosporine were used in combination with corticosteroids.

The HLH-2004 diagnostic criteria remain the most widely accepted international standard for diagnosing HLH [18]. However, these guidelines were developed primarily for pediatric patients with primary HPS and have several limitations when applied to adults. These include the exclusion of malignancy-associated HPS and diagnostic thresholds that may not be appropriate for adult-onset cases. As a result, alternative diagnostic frameworks have been proposed for adults. Tsuda’s criteria require the presence of all the following: a persistent high fever lasting more than one week, cytopenia affecting at least two hematopoietic lineages, and evidence of hemophagocytosis in the bone marrow or other tissues [19]. In this case, all three of Tsuda’s criteria were fulfilled, although no definitive underlying etiology was identified. Although the underlying etiology of HLH was not definitively identified, comprehensive investigations for infectious, malignant, and autoimmune triggers were negative. Based on the clinical presentation, a preceding viral illness was suspected to be the most likely trigger.

In cases of acute liver dysfunction with coagulopathy during pregnancy, the differential diagnosis is extensive. Other major causes, such as sepsis, viral hepatitis, and microangiopathic hemolytic anemias (including HELLP syndrome and TMA), were excluded based on clinical and laboratory findings. Intrahepatic cholestasis of pregnancy (ICP) was also considered; however, the presence of persistent high fever, progressive pancytopenia, and severe coagulopathy was inconsistent with the typical presentation of ICP. Furthermore, posterior reversible encephalopathy syndrome was also considered unlikely due to the absence of hypertension and characteristic neurological symptoms [20]. The primary diagnostic challenge, therefore, centered on distinguishing HLH from AFLP.

A definitive diagnosis of AFLP is established by liver biopsy demonstrating microvesicular fatty infiltration within hepatocytes. However, in patients with severe coagulopathy, liver biopsy is often not feasible [21]. The Swansea criteria, which consist of 14 clinical, pathological, and radiological findings, are commonly used to support the diagnosis of AFLP. The Swansea criteria are positive when at least six out of 14 items are fulfilled and have demonstrated a positive predictive value of 85% and a negative predictive value of 100% in previous studies [22]. In our case, the patient met six criteria: abdominal pain, elevated bilirubin, elevated urate, elevated transaminases, coagulopathy, and a bright liver on imaging (Table 3).

**Table 3:** Swansea criteria AST: Aspartate aminotransferase, ALT: Alanine aminotransferase, Cre: Creatinine, PT: Prothrombin time, APTT: Activated partial thromboplastin time.

Class	Variable	Finding
Clinical features	1. Vomiting	-
	2. Abdominal pain	+
	3. Polydipsia/polyuria	-
	4. Encephalopathy	-
Laboratory features	1. Elevated bilirubin (> 0.8 mg/dL)	+
	2. Hypoglycemia (< 72 mg/dL）	-
	3. Elevated urate (> 5.7 mg/dL)	+
	4. Leucocytosis (> 11000 /μL)	-
	5. Elevated AST/ALT (> 42 U/L)	+
	6. Renal impairment (Cre > 1.7 mg/dL)	-
	7. Elevated anmonia (> 27.5 mg/dL or > 47 μmol/L)	-
	8. Coagulopathy (PT > 14 sec or APTT > 34 sec)	+
Radiographic features	Bright liver/ascites	+
Histologic features	Microvesicular steatosis	-
Total variables	14	6

Although the patient satisfied the Swansea criteria, the presence of pancytopenia, hepatosplenomegaly, and hemophagocytosis on bone marrow examination necessitated consideration of HLH. A crucial point for differentiation was the clinical course after delivery; maternal conditions typically improve after delivery in AFLP, but our patient’s condition worsened, which strongly suggested HLH [23]. Given the coagulopathy, we initiated PE therapy and transfused 8 units of fresh frozen plasma (FFP) before performing an ultrasound-guided liver biopsy. Histological analysis revealed predominantly large lipid droplets without the characteristic microvesicular steatosis typically seen in AFLP.

Disorders of fatty acid β-oxidation have been associated with mutations in the long-chain-3-hydroxyacyl CoA dehydrogenase (LCHAD) gene. Inheritance of mutated alleles from both parents impairs fetal fatty acid metabolism, leading to the accumulation of abnormal metabolites. These metabolites can cross the placenta and accumulate in the maternal circulation, ultimately inducing lipotoxicity in the maternal liver [24]. In this patient, genetic analysis revealed no mutations in the LCHAD gene. Furthermore, newborn screening of the second child was negative for LCHAD deficiency. Although clinical findings suggested the possibility of AFLP, the diagnosis was ultimately excluded based on liver biopsy and genetic analysis.

Although no standardized scoring system exists for determining liver transplantation eligibility in pregnancy-related acute liver failure, the BILE score, which incorporates bilirubin, lactate, and etiology, has been proposed as a useful prognostic tool [25]. In our case, the BILE score was 7.43, which exceeded the proposed cutoff of 6.9. Liver transplantation was therefore considered. However, due to the insufficient response to high-dose steroids, cyclosporine was initiated, resulting in hematological improvement and obviating the need for liver transplantation.

During the ICU stay, the patient experienced two episodes of life-threatening hemorrhage. The first occurred after liver biopsy due to intra-abdominal bleeding; the second was caused by genital bleeding associated with hypofibrinogenemia. HLH is commonly accompanied by coagulopathy, with hypofibrinogenemia being the most frequently observed abnormality. Although the exact mechanism remains unclear, excessive activation of the fibrinolytic system, including elevated plasmin levels, has been proposed [26]. In this case, laboratory findings after ICU admission showed significant fibrinolytic activation: fibrinogen 98 mg/dL, FDP 54.6 μg/mL, D-dimer 46.2 μg/mL, plasmin α2-plasmin inhibitor complex (PIC) 16.4 μg/mL, and thrombin antithrombin Ⅲ complex (TAT) 114 ng/mL. In retrospect, direct visualization during liver biopsy might have reduced the risk of hemorrhage in such a coagulopathic patient. During the second hemorrhagic episode, we administered 8 units of FFP, 3 g of fibrinogen concentrate, and 150 mL of cryoprecipitate (equivalent to 12 units of FFP at our institution). These interventions successfully increased the fibrinogen level to 178 mg/dL. Maintaining fibrinogen levels above 150 mg/dL is recommended for managing massive hemorrhage, and both fibrinogen concentrate and cryoprecipitate are effective for rapid correction [27]. In this case, circulatory stabilization after fibrinogen replacement likely reflected the efficacy of these therapies.

## Conclusions

We experienced a rare case of HLH diagnosed after a cesarean section, which presented considerable diagnostic challenges due to its clinical overlap with AFLP. Because AFLP can rapidly progress to liver failure requiring liver transplantation, differentiating between these two conditions is crucial. In cases of significant coagulopathy associated with HLH, the timing and approach of invasive diagnostic procedures such as liver biopsy should be carefully considered to minimize the risk of hemorrhagic complications.
